# Treatment with the Antipsychotic Agent, Risperidone, Reduces Disease Severity in Experimental Autoimmune Encephalomyelitis

**DOI:** 10.1371/journal.pone.0104430

**Published:** 2014-08-12

**Authors:** David O'Sullivan, Laura Green, Sarrabeth Stone, Pirooz Zareie, Marie Kharkrang, Dahna Fong, Bronwen Connor, Anne Camille La Flamme

**Affiliations:** 1 School of Biological Sciences, Victoria University of Wellington, Wellington, New Zealand; 2 Department of Pharmacology and Clinical Pharmacology, Centre for Brain Research, Faculty of Medical and Health Sciences, University of Auckland, Auckland, New Zealand; 3 Malaghan Institute of Medical Research, Wellington, New Zealand; Washington University, United States of America

## Abstract

Recent studies have demonstrated that atypical antipsychotic agents, which are known to antagonize dopamine D2 and serotonin 5-HT_2a_ receptors, have immunomodulatory properties. Given the potential of these drugs to modulate the immune system both peripherally and within the central nervous system, we investigated the ability of the atypical anti-psychotic agent, risperidone, to modify disease in the animal model of multiple sclerosis (MS)^4^, experimental autoimune encephalomyelitis (EAE). We found that chronic oral administration of risperidone dose-dependently reduced the severity of disease and decreased both the size and number of spinal cord lesions. Furthermore, risperidone treatment substantially reduced antigen-specific interleukin (IL)-17a, IL-2, and IL-4 but not interferon (IFN)-γ production by splenocytes at peak disease and using an *in vitro* model, we show that treatment of macrophages with risperidone alters their ability to bias naïve T cells. Another atypical antipsychotic agent, clozapine, showed a similar ability to modify macrophages *in vitro* and to reduce disease in the EAE model but this effect was not due to antagonism of the type 1 or type 2 dopamine receptors alone. Finally, we found that while risperidone treatment had little effect on the *in vivo* activation of splenic macrophages during EAE, it significantly reduced the activation of microglia and macrophages in the central nervous system. Together these studies indicate that atypical antipsychotic agents like risperidone are effective immunomodulatory agents with the potential to treat immune-mediated diseases such as MS.

## Introduction

MS is a chronic inflammatory disease, mediated by immune cells targeting the myelin sheaths surrounding the nerve axons and is characterized by plaque formation and central nervous system (CNS) dysfunction. The symptoms of MS are mainly related to altered nerve conduction, but there is much heterogeneity in disease presentation and pathology; for example, disease symptoms can be chronic or transient and range from mild to severe [1], [2]. The diversity in disease symptoms, clinical progression and the immune responses that underlie the clinical presentation, adds to the complexity of both diagnosing and treating MS.

There are currently ten FDA approved disease-modifying agents, which are mainly used in the treatment of relapsing–remitting MS. Of these drugs, only three are orally administered, and none are effective for primary-progressive disease [3]. While all MS therapies have distinct mechanisms of actions, most have a limited efficacy rate of between 30–60% or are associated with significant side-effects or toxicity (e.g. natalizumab, mitoxantrone) [4], [5], [6]. Thus, there is a great need for alternative therapies with greater efficacy, which target forms of MS for which there are no effective treatments, or that cause minimal side effects when chronically administered.

Atypical antipsychotic agents such as risperidone and clozapine are used to treat schizophrenia and other psychiatric disorders, although recent research suggests these compounds may also have the potential to modulate the immune system [7], [8], [9]. The pathophysiology of schizophrenia is complex and can involve alterations in brain structure as well as a dysregulation of signalling by a number of neurotransmitters, such as dopamine and serotonin [10], [11] Atypical antipsychotic agents block dopamine and serotonin from binding to their cognate receptors and it is through this process that antipsychotics are thought to exert their main therapeutic effects [12], [13]. However, as many of these agents can also bind to and antagonize a variety of dopamine, serotonin, histamine and adrenergic receptors at low nanomolar affinities, identification of a distinct mechanism of action is difficult [14], [15]. Additionally, schizophrenia is often associated with alterations in immune parameters, and it has been hypothesized that some of the therapeutic benefits of anti-psychotic medication may be associated with immune-modulation [16], [17], [18]. Supporting this concept, a number of studies have found that atypical antipsychotic drugs cause alterations in serum cytokine and cellular immune responses within schizophrenic patients [19], [20], [21]. Furthermore it has been demonstrated that atypical antipsychotic agents such as risperidone, clozapine and olanzapine have anti-inflammatory effects *in vitro* as well as *in vivo* in animal models of inflammation [7], [9], [22], [23].

Given the evidence suggesting that atypical antipsychotic agents have the potential to modulate the immune system both peripherally and within the CNS, it is possible that these compounds may be of benefit to inflammatory diseases. To explore this possibility, we investigated if the atypical antipsychotic agent, risperidone, could modify disease expression in an animal model of MS, EAE.

## Materials and Methods

### Animals and Reagents

Female C57BL/6 mice were purchased from the Biomedical Research Unit of the Malaghan Institute for Medical Research (Wellington, NZ), and 2D2 mice, which express a transgenic TCR for the myelin oligodendrocyte glycoprotein (MOG)_35–55_ peptide (originally from the Malaghan Institute of Medical Research) [24], were bred at Victoria University of Wellington. Both strains were used between 8–12 weeks of age. Food and water were available *ad libitum*, and all experimental procedures used in this study were approved by the Victoria University of Wellington Animal Ethics Committee (approval 2008-R12).

MOG_35–55_ peptide was purchased from Genescript (Piscataway, NJ). Heat-killed *Mycobacterium tuberculosis* H37Ra was purchased from Difco (Lawrence, KS), incomplete Freund's adjuvant from Sigma-Aldrich (St. Louis, MO), and pertussis toxin from List Biochemical (Campbell, CA). Risperidone and clozapine were kindly supplied by Douglas Pharmaceuticals Ltd. (Auckland, New Zealand) and were dissolved in 0.1 M acetic acid to a stock concentration of 6 mg/ml then diluted to the required final concentration using autoclaved tap water. To achieve correct daily dosages, average daily water consumption was measured before initiation of experiments, and the correct dosage of the drugs were calculated accordingly.

### Induction of experimental autoimmune encephalomyelitis and lesion analysis

EAE was induced by s.c. immunization in the rear flanks of mice with MOG peptide (50 µg/mouse) emulsified in complete Freund's adjuvant (500 µg/mouse *M. tuberculosis*). Pertussis toxin (200 ng/mouse) was injected i.p. on days 0 and 2. Mice were weighed and scored daily for any signs of disease by a non-blinded investigator, using the following disease rating scale: 0, normal; 1, partial tail paralysis; 2, full tail paralysis; 3, full paralysis of one hind limb; 4, full paralysis of both hind limbs; and 5, moribund. At euthanasia, brains and spinal cords were removed, fixed with zinc salts or 4% paraformaldehyde, and paraffin-embedded for histological analysis. Lesion size and number were assessed on hematoxylin and eosin-stained tissue sections and expressed per 100,000 µm^2^. All histological analyses were done in a blinded fashion.

### Isolation and *in vitro* culture of cells

Spleens were isolated at euthanasia and single cell suspensions made by passage through a cell strainer (70 µm; BD Biosciences, Franklin Lakes, NJ). After lysing red blood cells with Red Cell Lysis Buffer (Sigma-Aldrich), cells were washed and resuspended in complete culture medium containing DMEM, 10% FCS, 100 U/ml penicillin plus 100 µg/ml streptomycin, 10 mM Hepes, 2 mM L-glutamine, and 50 µM 2-ME (all from Life Technologies, Carlsbad, CA). Splenocytes (10^6^ cells/well) were stimulated with MOG peptide (50 µg/ml), ConA (3 µg/ml; Sigma-Aldrich), or lipopolysaccharide (LPS; 200 ng/ml; Sigma-Aldrich) in 96 well plates and the supernatants were removed for analysis 72, 48, or 24 hours later, respectively.

Bone marrow-derived macrophages (BMMΦ) were isolated and cultured as described [25], and RAW264.7 cells were kindly gifted from the Malaghan Institute of Medical Research and used at an early passage number. RAW264.7 cells (5×10^4^/well) and BMMΦ (10^5^/well) were cultured in complete culture medium in 96-well plates and primed overnight with IFN-γ (20 U/ml; Peprotech, Rocky Hill, NJ) before stimulating with or without LPS (200 ng/ml) and risperidone, clozapine, sulpiride (Sigma-Aldrich), LE300 (R&D Systems, Minneapolis, MN), L741,626 (R&D Systems), and dopamine (Sigma-Aldrich) as indicated.

### Macrophage-T cell co-cultures

CD4^+^ T cells were isolated from spleens of 2D2 using CD4 (L3T4) Dynabeads (Life Technologies) according to manufactures instructions. BMMΦ (1×10^5^/well) were primed with IFN-γ (20 U/ml) overnight, following which IFN-γ was removed by washing with warm medium and the BMMΦ were stimulated as described. Four hours following BMMΦ stimulation CD4^+^ T cells (2.5×10^5^/well) and MOG (25 µg/ml) were added to the BMMΦ cultures and incubated for 72 hours. In some instances the stimuli were removed by washing with warm medium prior to the addition of T cells and MOG.

### Cytokine and viability assays

Cytokine levels in splenocyte culture supernatants were assessed by the Th1/Th2/Th17/Th22 13-plex cytokine bead array (CBA) kit (Bender MedSystems) according to the manufacturer's instructions. IL-12p40 and IL-10 in macrophage culture supernatants were measured by ELISA as previously described [26], and all reagents were purchased from BD Biosciences. The MTT assay was performed as described to determine the effect of the treatments on viability based upon changes to cellular metabolism [27]. The Griess reaction was used to assess NaNO_2_ levels in macrophage culture supernatants as described [28]. Dopamine in macrophage culture supernatants was measured by HPLC as described [29].

### Immunohistochemistry

Mice were euthanized and transcardially perfused with PBS, followed by 4% paraformaldehyde. Brains and spinal cords were removed and post-fixed in 4% paraformaldehyde after which spinal cord samples were embedded in paraffin and brains cryoprotected in 30% (w/v) sucrose/PBS in preparation for sectioning.

Immunohistochemistry was performed on 7 µm coronal spinal cord sections and 30 µm sagittal free-floating brain cryosections using antibodies against the macrophage/microglial markers: goat anti-Iba-1 pAb (1∶750; ab5076, Abcam, Cambridge, MA) and biotinylated rat anti-F4/80 mAb (1∶100; MCA497B, Serotec, Kidlington, UK). Following endogenous peroxidase blocking the sections were incubated overnight in primary antibodies diluted in immunobuffer (PBS containing 4% horse serum and 0.2% Triton X-100). For Iba-1 labeling sections were incubated with biotinylated secondary antibody diluted in immunobuffer (1∶1000; Jackson ImmunoResearch, West Grove, PA) for 2–3 hr. Bound antibody was detected using the Vectastain Elite ABC kit (Vector Laboratories, Burlingame, CA) with the VIP substrate (Vector Laboratories). Slides were counterstained with hematoxylin (Sigma-Aldrich).

The level of immunostaining in each CNS region was assessed by an investigator blinded to treatment. Two scores of 0–3 were allocated for (1) level of marker expression and (2) level of focal accumulation of activated microglia. The sum of both these scores generated a total score of 0–6. Six spinal cord sections sampled at 70 µm intervals and three serial brain sections sampled at 720 µm intervals through the cerebellum, hippocampus, brain stem and olfactory bulb were evaluated for each mouse. See Figure S1 for scoring details and examples.

### Flow cytometric analyses

Expression of subset and activation markers on splenocytes was assessed using the following antibodies: rat anti-CD45, rat anti-CD4, rat anti-B220, rat anti-CD8, rat anti-CD11b, rat anti-Gr-1, and rat anti-FoxP3 from BD Biosciences (San Jose, CA) and rat anti-CD25, rat anti-CD14, and hamster anti-CD11c from BioLegend (San Diego, CA). Rat anti-F4/80 antibody was purchased from eBioscience (San Diego, CA). Fluorescently-labeled isotype control antibodies were matched to each specific antibody as recommended by the manufacturer. Additionally, polyclonal rabbit anti-dopamine receptor 1 (D1) and 2 (D2) antibodies were from Calbiochem (Merck, Darmstadt, Germany) and detected with PE-labeled goat anti-rabbit antibodies (Jackson Laboratories). Single cell suspensions were prepared as previously described (see: *Isolation and in vitro culture of cells*). Data was collected on a FACSCanto II (Becton Dickinson, Franklin Lakes, NJ) or FACScan (Becton Dickinson) and analyzed using FlowJo (Tree Star Inc., Ashland, OR). Live cells were gated using FSC and SSC before analysis of all splenocyte subsets except Tregs. For Treg identification, CD4 T cells were gated first using CD4 and FSC, and then CD25^+^CD4^+^ cells were selected. The identity of the Tregs was confirmed by FoxP3 staining (Figure S2). For D1 and D2 expression, after gating for live cells by FSC and SSC, fully differentiated macrophages were identified as CD11b^high^ and F4/80^high^.

### Statistical analyses

Data were analyzed by one-way ANOVA, two-way ANOVA, or Student's t test as indicated in the figure legend or text and using Prism software (GraphPad, La Jolla, CA). If p<0.05 by one-way ANOVA, post-tests were performed to determine which groups were significantly different. The Newman-Keul's multiple comparison test was used to assess all groups while the Dunnett's test was used to compare to a control condition. The Mann-Whitney test was used for non-parametric analyses between two groups and Kruskal-Wallis for non-parametric analyses with three or more groups. Differences of p<0.05 were considered significant.

## Results

### Treatment of mice with risperidone reduced disease severity and lesions in the spinal cord during EAE but did not alter disease incidence

**Figure 1:**
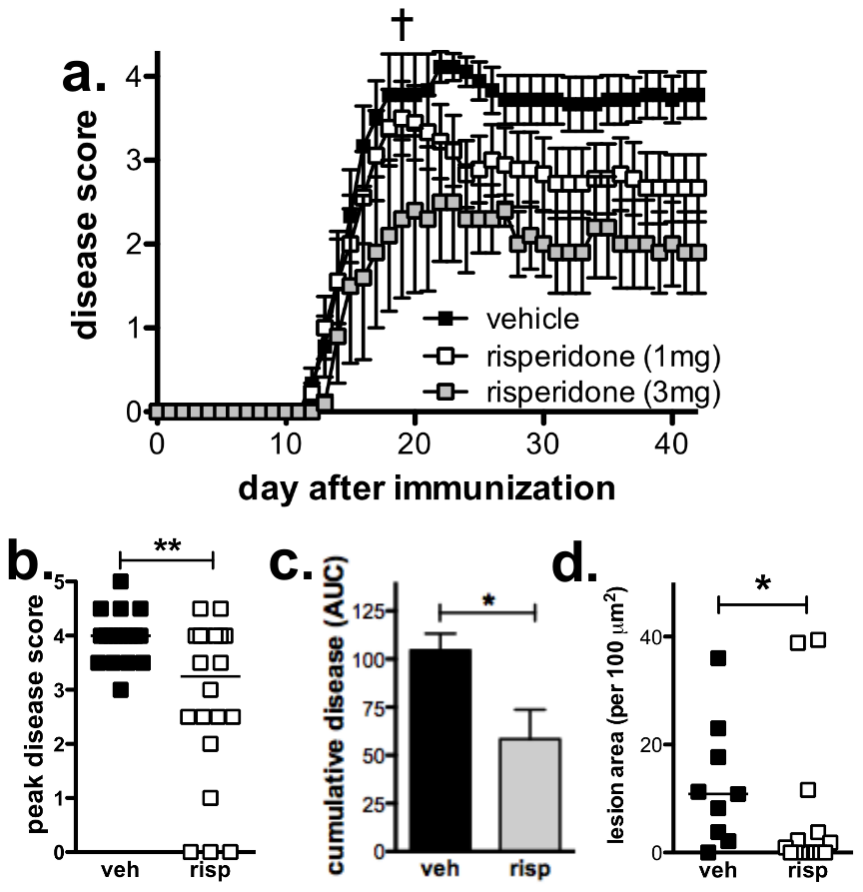
Risperidone dose-dependently reduces the severity of EAE. **a.** Mice were treated with risperidone (1 or 3 mg/kg/day; n = 10 and 5, respectively) or vehicle (n = 9) in their drinking water from the time of immunization and scored daily (0; normal – 5; moribund). Shown are the means and SEM of individual mice. Two mice from the vehicle group were euthanized on day 19 (indicated by †) and were excluded from analysis. p<0.05 by two-way ANOVA (vehicle vs risperidone 3 mg). **b.** Mice treated with 3 mg/kg/day risperidone (n = 20) have significantly reduced peak disease score compared to vehicle-treated mice (n = 18). Shown are the medians and values of individual mice from 4 experiments. **p = 0.01 by Mann-Whitney test. **c.** Risperidone (3 mg/kg/d) significantly reduces cumulative disease as assessed by the area under the curve (AUC) of individual animals 42 days post-immunization. Shown are the means and SEM of individual mice (n = 9 vehicle-treated and 5 risperidone-treated) from 2 experiments. *p<0.05 by unpaired Student's t test. **d.** Risperidone (3 mg/kg/day) reduces lesion area in the spinal cords of mice. Spinal cord lesions were assessed on H & E stained tissue (see Figure S5 for images) and expressed as lesion area per 100 µM area of tissue examined. Shown are the medians and values of individual mice (n = 9 vehicle and 15 risperidone-treated) from three experiments. *p<0.05 vehicle versus drug by Mann-Whitney test.

To investigate if the atypical anti-psychotic agent, risperidone, is effective at modifying the course of disease in the EAE mouse model, mice were immunized to induce EAE and treated daily with risperidone or the vehicle alone. The doses of risperidone (1 or 3 mg/kg/day) were chosen to be comparable to clinically relevant doses used in humans as estimated via comparable D_2_ receptor occupancy [30], [31], [32]. To achieve a prolonged drug exposure, mice were administered risperidone in their drinking water throughout the course of the experiments. No alteration in water consumption was observed between risperidone- or vehicle-treated animals and chronic administration did not cause any obvious adverse effects aside from modest weight gain in risperidone-treated, unimmunized mice as reported previously [33]. Risperidone-treated, immunized mice exhibited reduced disease severity compared to vehicle-treated, immunized mice (Figure 1a), and this reduction was particularly pronounced in the 3 mg/kg/day treatment group.

Risperidone treated mice also had reduced peak disease scores (Figure 1b) and cumulative disease, as assessed by the area under the curve (AUC; Figure 1c). In contrast to the disease severity, the incidence was similar (100% WT; 85% risperidone 3 mg/kg/day; and 100% risperidone 1 mg/kg/day), and the day of disease onset modestly but significantly delayed in risperidone-treated compared to vehicle-treated, immunized animals (11.6±0.5, 13.4±0.6, and 15.1±1.1*; vehicle, risperidone 1 and 3 mg/kg/day respectively; *p<0.05 as assessed by Kruskal-Wallis test with Dunn's Multiple Comparison test). Because CNS lesions occur primarily in the spinal cord during the acute stage of EAE in C57BL/6 mice, we assessed lesion development in the spinal cord [34]. As shown in Figure 1d (see Figure S3 for images), risperidone treatment led to a reduction in the total lesion area, and a similar reduction in the number of lesions was also observed (7.0±1.4 per 100,000 µm^2^ compared to 2.75±1.9; vehicle vs risperidone). Together these results indicate that risperidone is effective at reducing disease when chronically administered to mice.

### Risperidone treatment correlated to an altered antigen-specific Th cytokine balance but does not prevent development of antigen-specific responses

To identify if the attenuated disease response in risperidone-treated animals correlated with alterations in the immune compartment, the cellular composition and antigen-specific recall responses in peripheral lymphoid organs were examined. On day 15 when disease peaked in the vehicle-treated animals, no difference was observed in the total number of splenocytes or in the total number of CD8 T cells or Tregs between any of the groups (Table 1). Although immunization overall caused a decrease in the total number of CD4 T cells and B cells, risperidone treatment itself had no effect (Table 1). By the chronic phase of disease (day 40), there were no differences in CD8 T cells or B cells whereas the number of Tregs and CD4 T cells was significantly increased by risperidone treatment of immunized mice (Table 1; Figure S2a). These results suggest that while immunization induced clear changes in the lymphoid compartment at peak disease, risperidone treatment did not. In contrast, during the chronic phase of disease, risperidone enhanced the number of splenic Tregs and CD4 T cells.

**Table 1:** Effect of risperidone treatment on splenocyte subpopulations.

Treatment	Day after immunization	Total splenocytes (×10^7^)	Total B cells (×10^7^)	Total CD8 T cells (×10^6^)	Total CD4 T cells (×10^7^)	Total Tregs (×10^6^)
Unimmunized, vehicle-treated	15	8.9 +/- 1.0	6.1 +/- 0.6	5.3 +/- 1.4	1.8 +/- 0.2	1.4 +/- 0.3
Unimmunized, risperidone-treated	15	9.6 +/- 1.0	6.1 +/- 0.6	4.9 +/- 1.0	1.7 +/- 0.2	1.3 +/- 0.3
Immunized, vehicle-treated	15	6.5 +/- 0.9	2.6 +/- 0.3**	3.3 +/- 0.7	0.9 +/- 0.1**	0.6 +/- 0.1
Immunized, risperidone-treated	15	9.6 +/- 1.4	3.3 +/- 0.6**	6.1 +/- 1.2	1.2 +/- 0.1	0.8 +/- 0.2
Unimmunized, vehicle-treated	40	5.4 +/- 1.0	2.7 +/- 0.8	0.6 +/- 0.1	0.7 +/- 0.1	1.2 +/- 0.5
Unimmunized, risperidone-treated	40	6.3 +/- 0.9	2.8 +/-0.5	0.9 +/- 0.2	1.0 +/- 0.2	1.3 +/- 0.3
Immunized, vehicle-treated	40	9.5 +/- 1.2	3.0 +/- 0.5	0.7 +/- 0.1	1.4 +/- 0.2*	1.7 +/- 0.2*
Immunized, risperidone-treated	40	8.7 +/- 1.0	3.1 +/- 0.5	0.9 +/- 0.1	2.4 +/- 0.4**	3.0 +/- 0.4**

* p<0.05 by one-way ANOVA with Newman-Keul's multiple comparison test; vehicle, immunized compared to risperidone, immunized at that time point.

** p<0.05 by one-way ANOVA with Newman-Keul's multiple comparison test; immunized compared to unimmunized at that time point.

**Figure 2:**
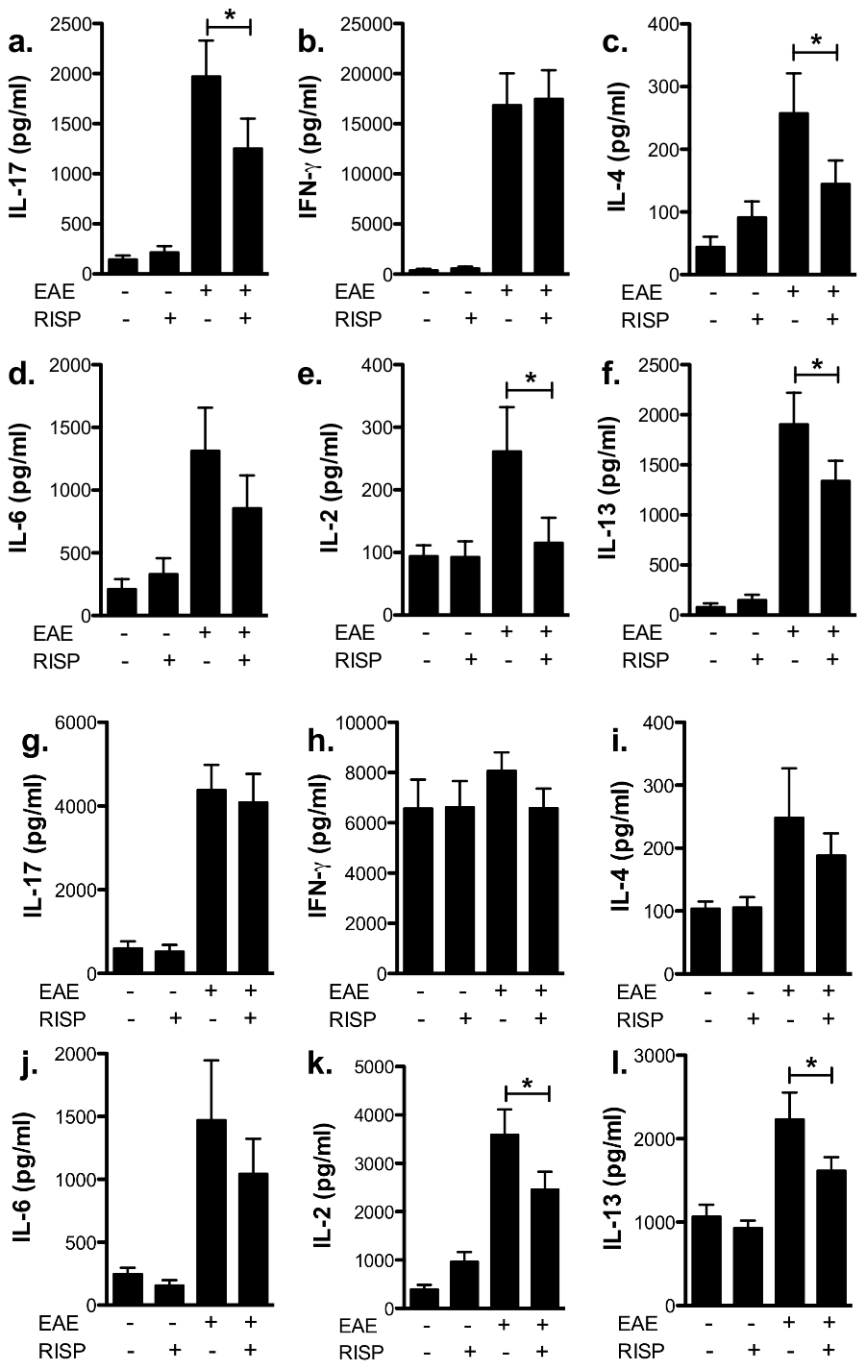
Risperidone treatment sustains antigen-specific IFN-γ responses and depresses IL-17a and IL-4 responses in EAE-immunized mice. Splenocytes were isolated from risperidone- and vehicle-treated, unimmunized and immunized mice 15 days post-immunization and stimulated *in vitro* with MOG peptide for 72 hours (a-f) or ConA for 48 hours (g-l). IL-17a (a and g), IFN- γ (b and h), IL-4 (c and i), IL-6 (d and j), IL-2 (e and k), IL-13 (f and l) production from individual mice was assessed by CBA. Shown are the means and SEM of individual mice from three experiments (n = 10–15 per group). * p<0.05 by one-way ANOVA with Newman-Keul's multiple comparison test.

Although risperidone-treated, immunized mice had similar numbers of CD4 T cells compared to vehicle-treated, immunized mice at peak disease, the cytokine profile of these cells was altered. A significant decrease in the production of IL-17a, IL-2, IL-4, and IL-13 occurred in MOG-stimulated splenocytes from risperidone-treated when compared to vehicle-treated mice during the peak of disease, whereas IFN-γ production was unaffected by risperidone treatment (Figure 2a-f). Splenocytes from unimmunized mice did not produce any antigen-specific cytokines as expected (Figure 2a-f), and polyclonal stimulation of T cells with concanavalin A (ConA) resulted in a similar reduction in IL-2 and IL-13 but not IL-17a or IL-4 (Figure 2g–l). Interestingly, in the chronic phase of disease, we found no difference in MOG-specific IL-17a or IL-10 production but instead observed a significant increase in IFN-γ (Figure S2b–d). Taken together, these findings indicate that antigen-specific T cell responses are differentially modulated during the acute and chronic phases of EAE by risperidone treatment.

### Risperidone treatment of bone marrow-derived macrophages (BMMΦ) reduced IL-12p40, enhanced IL-10 production, and altered their ability to bias naïve T cells

This research raises the intriguing possibility that the antipsychotic agent, risperidone, could be beneficial for the treatment of MS, but the mechanism by which it modulates encephalitogenic immune responses is unclear. Because previous studies have shown that risperidone and other antipsychotic compounds can reduce the production of inflammatory cytokines such as IFN-γ, IL-1β, IL-6 and TNF-α production by microglia (CNS resident macrophage-like cells), we first investigated whether BMMΦ, like microglia, expressed dopaminergic receptors or secreted dopamine *in vitro* [8], [22], [35], [36]. In agreement with other studies, we found that BMMΦ expressed low levels of dopamine receptor 1 (D1) and 2 (D2; Figure S4a) and produced endogenous dopamine under *in vitro* culture conditions (279 ng/ml or ∼1.5µM) indicating that BMMΦ can produce and respond to endogenous dopamine [37], [38], [39].

**Figure 3:**
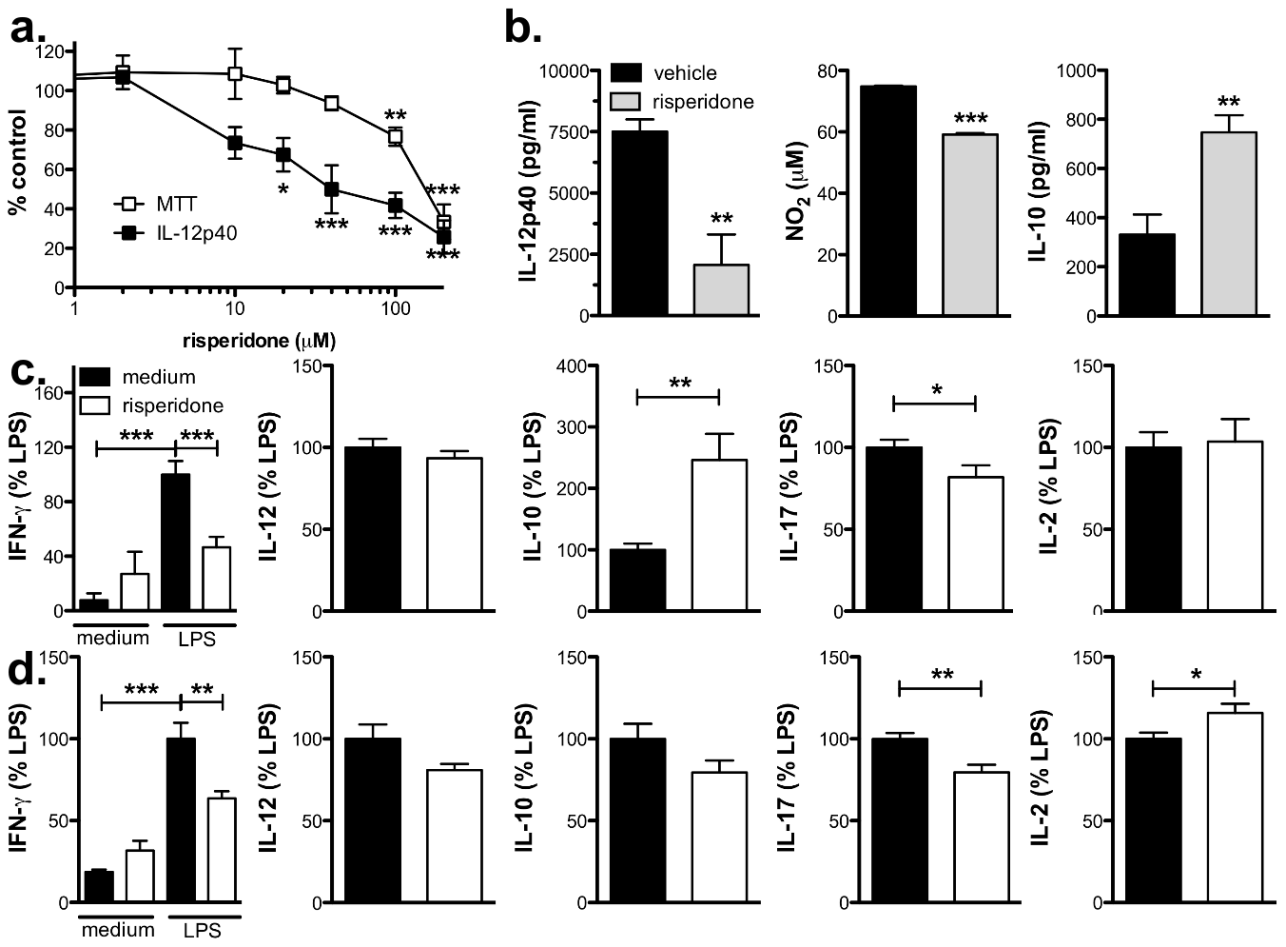
Risperidone reduces IL-12p40 and nitric oxide production and enhances IL-10 production by LPS-stimulated BMMΦ (a and b) and modifies their ability to bias CD4 T cells (c and d). **a.** Risperidone inhibited IL-12p40 production by LPS-stimulated BMMΦ in a concentration-dependent manner and at high concentrations affected viability as assessed after 24 hours by MTT assay. Values are expressed as % control (i.e. compared to LPS + vehicle), and shown are the means and SEM of values from 5 experiments. *p<0.05, **p<0.01, and ***p<0.001 by one-way ANOVA with Dunnett's post-test (compared to LPS + vehicle). **b.** Risperidone (20 µg/ml) significantly reduced IL-12p40 and NO but enhanced IL-10 production by LPS-stimulated BMMΦ. Shown are the means and SEM of replicate wells from 1 of at least 3 experiments (IL-12p40 and NO) or combined from 2 experiments (IL-10). **p<0.01 and ***p<0.001 by unpaired Student's t test. **c & d**. LPS-stimulated BMMΦ activated purified MOG-specific 2D2 T cells and 4 hour pretreatment (**d**) or continuous exposure (**c**) of BMMΦ to risperidone (50 µM) for 4 hour before addition of T cells modified T cell biasing as assessed by IFN-γ, IL-17a, and CD124 expression. Shown are the means and SEM of triplicate wells from 4 (**c**) or 2 (**d**) experiments. *p<0.05, **p<0.01, and ***p<0.001 by one-way ANOVA with Newman-Keul's post test (IFN-γ) or by paired Student's t test (IL-12, IL-10, IL-17a, IL-2, and CD124).

To determine if risperidone had direct immunomodulatory effects on BMMΦ, we simulated an inflammatory environment *in vitro* by priming with IFN-γ followed by stimulation with LPS in the presence or absence of risperidone during the culture period. To ensure that risperidone did not have an adverse effect on macrophage viability, an MTT assay was performed, and while high concentrations of risperidone (≥100 µM) resulted in a substantial decrease in MTT metabolism compared to the vehicle-treated control cultures, concentrations below 100 µM did not significantly alter the metabolic activity of LPS-stimulated macrophages (Figure 3a). In comparison, the production of the inflammatory cytokine IL-12 was attenuated in a concentration-dependent manner at doses ≥20 µM (Figure 3a and b). LPS-stimulated NO production was also modestly but significantly reduced by risperidone whereas the regulatory cytokine, IL-10, was increased by risperidone treatment (Figure 3b). None of these cytokines or mediators was induced by risperidone alone indicating that this drug is acting by modifying existing inflammatory pathways (data not shown).

Given that we found alterations in antigen-specific cytokine production in risperidone-treated mice during EAE, we investigated whether the direct effect of risperidone on BMMΦ cytokine production would lead to an altered ability of BMMΦ to activate and bias naïve T cells in an antigen-specific manner. Using IFN-γ-primed, LPS-stimulated BMMΦ to present the MOG peptide to purified CD4 T cells from 2D2 mice, which express the MOG-specific TCR, we found that LPS stimulation of BMMΦ led to an induction of strong Th1 (i.e. IFN-γ) and relatively weak Th17 responses (Figure S4b) whereas in the absence of LPS stimulation only low levels of cytokines were detected (Figure 3c and Figure S4b). The continuous presence of risperidone in this *in vitro* model of T cell activation shifted phenotype of the T cells by reducing IFN-γ and IL-17a production and decreasing the expression of CD124 (i.e. IL-4Rα; Figure 3c and Figure S4c) but did not alter T cell activation as assessed by IL-2, CD62L, and CD44 expression (Figure 3c and Figure S4c). While IL-12 levels were not affected, IL-10 production was significantly enhanced by the presence of risperidone (Figure 3c).

To verify that the changes were due to a direct effect of risperidone on BMMΦ, we pretreated BMMΦ with risperidone for 4 hours before removing the drug and adding purified CD4 T cells and antigen (Figure 3d). Even when only the BMMΦ had been exposed to risperidone, the levels of IFN-γ and IL-17a were similarly reduced (Figure 3d). Interestingly, IL-10 levels were not enhanced, and IL-2 levels were modestly but significantly increased. Although the lack of enhancement in IL-10 production may suggest that T cells are the source of the IL-10, because IL-10 is produced by LPS-stimulated macrophages in the presence of risperidone, it is likely that the 4 hour exposure to risperidone was not sufficient to enhance IL-10 in this system. Taken together, these studies indicate that risperidone has direct immunomodulatory effects on BMMΦ and these effects include a reduction in pro-inflammatory cytokine secretion as well as an altered ability to bias but not activate CD4 T cells.

### Treatment with another atypical antipsychotic also reduces EAE severity and inhibits CNS infiltration

**Figure 4:**
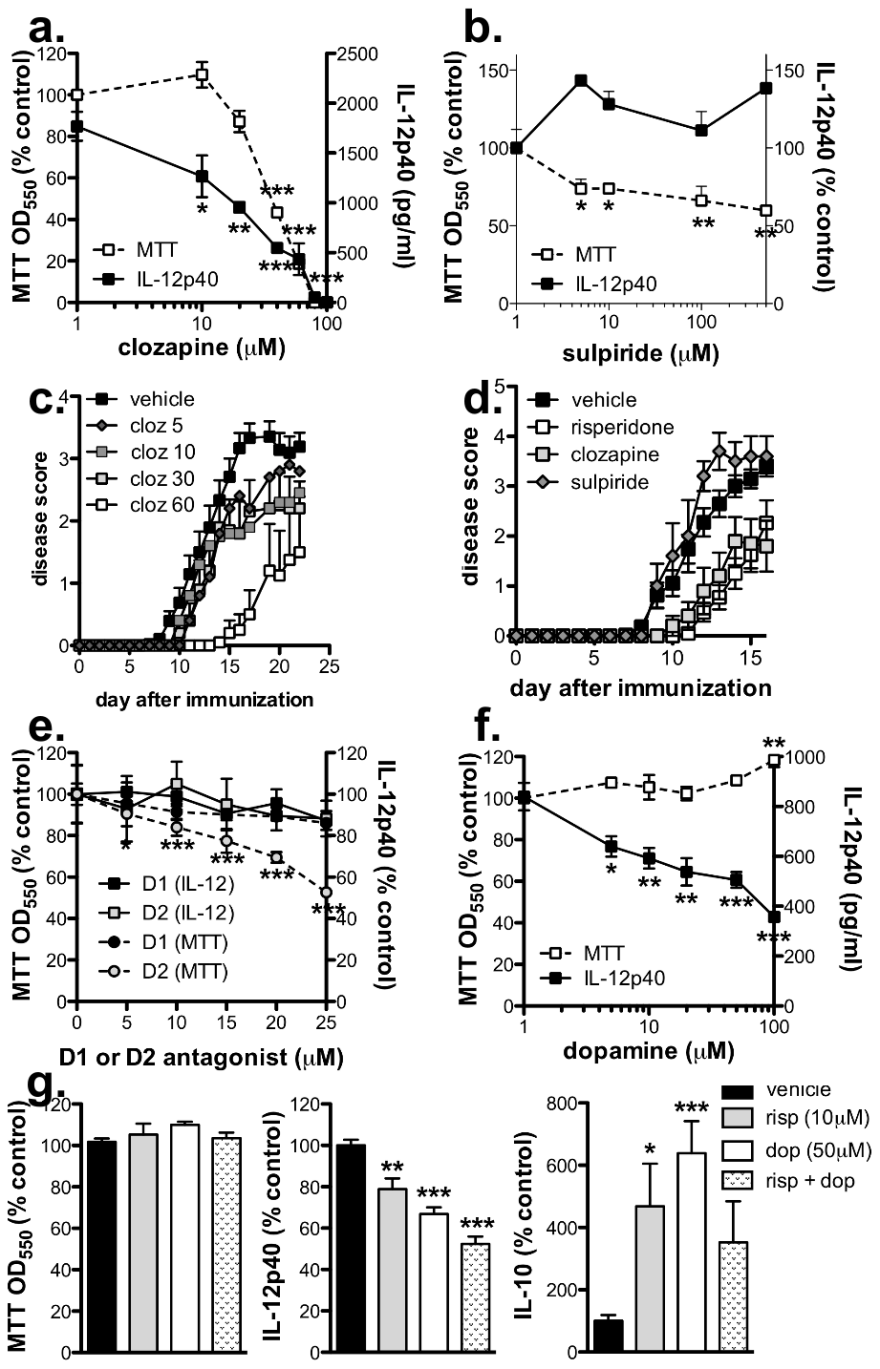
Another atypical antipsychotic agent, clozapine, also reduces the severity of EAE. **a & b.** Clozapine (**a**) but not sulpiride (**b**) inhibited IL-12p40 production by LPS-stimulated BMMΦ in a concentration-dependent manner and at higher concentrations affected viability as assessed after 24 hours by MTT assay. Values are expressed as % control (i.e. compared to LPS + vehicle), and shown are the means and SEM from 1 of 3 (**a**) or 2 (**b**) experiments. *p<0.05, **p<0.01, and ***p<0.001 by one-way ANOVA with Dunnett's post-test (compared to LPS + vehicle). **c.** Mice were treated with clozapine (5–60 mg/kg/day) or vehicle in their drinking water from the time of immunization and scored daily (0; normal – 5; moribund). Shown are the means and SEM of individual mice (n = 5 per group) from 1 of 4 dose-response experiments. **d.** Clozapine (30 mg/kg/day; n = 10) reduces EAE disease to a similar extent as risperidone (3 mg/kg/day; n = 20) but sulpiride (100 mg/kg/day; n = 5) is ineffective compared to vehicle treatment (n = 27). Shown are the means and SEM of individual mice from 4 experiments. **e.** Blocking dopamine signaling with a specific D1 antagonist (LE300) or D2 antagonist (L741,626) did not affect IL-12 production by RAW264.7 macrophages but D2 antagonism did reduce viability as assessed by MTT assay. Values are expressed as % control (i.e. compared to LPS + vehicle), and shown are the means and SEM of values from 1 of 2 experiments. *p<0.05, **p<0.01, and ***p<0.001 by one-way ANOVA with Dunnett's post-test (compared to LPS + vehicle). **f & g.** Dopamine inhibited IL-12p40 production by RAW264.7 macrophages without affecting viability (**f**) and risperidone enhanced the effects of dopamine on IL-12 but not IL-10 production (**g**). Shown are the means and SEM combined from 7 experiments. *p<0.05, **p<0.01, and ***p<0.001 by one-way ANOVA with Dunnett's post-test (compared to LPS + vehicle).

Risperidone like other atypical antipsychotic agents antagonizes a wide range of dopaminergic, serotonergic, muscanergic, and adrenergic receptors, yet it is the antagonism of D2 specifically that is believed to mediate the antipsychotic effects. To understand more fully which receptors are involved in mediating the effects of risperidone in the EAE model, we first investigated the effects of other anti-psychotic agents on IL-12 production by IFN-γ-primed, LPS-stimulated BMMΦ. We found that clozapine, which also antagonizes a wide range of neuroreceptors, but not sulpiride, a specific D2 antagonist, reduced IL-12 production in a concentration-dependent manner at levels ≥10 µM (Figure 4a and b). Although concentrations of clozapine ≥40 µM significantly reduced viability, sulpiride also significantly reduced viability without affecting IL-12 production. When these agents were administered to mice, clozapine but not sulpiride dose-dependently reduced EAE disease suggesting that the ability of these agents to modify BMMΦ responses *in vitro* correlates to their disease-reducing properties (Figure 4c and d). Moreover, the protective effect afforded by clozapine (30 mg/kg/day) was similar to risperidone (3 mg/kg/day) (Figure 4d) although a greater effect on disease was observed at higher doses of clozapine (60 mg/kg/day; Figure 4c). No adverse effects were observed in the unimmunized or immunized clozapine-treated groups except for a modest weight gain similar to that found in risperidone-treated mice (data not shown).

Because the antagonism of D2 by sulpiride did not appear to be responsible for the effects of risperidone on BMMΦ, we investigated whether preventing the action of dopamine signaling by a D1-specific antagonist was involved. Addition of the D1-specific antagonist LE300 did not affect the viability of RAW264.7 macrophages nor did it lead to a reduction in IL-12p40 production induced by LPS stimulation (Figure 4e). As found with sulpiride, the D2 specific antagonist L741,626 did not affect LPS-stimulated IL-12p40 production but did cause a reduction in viability at concentrations ≥5 µM (Figure 4e). Interestingly, we found that the addition of dopamine itself to the macrophage cultures concentration-dependently reduced IL-12p40 production and even increased cellular viability at high concentrations (Figure 4f). Moreover, if dopamine and risperidone were combined *in vitro*, together they resulted in a more significant decrease in IL-12p40 production without altering viability or IL-10 production (Figure 4g-i). Similar effects were found using BMMΦ exposed to LE300, L741,626, and dopamine (data not shown). Taken together, these results suggest that the immunomodulatory effects of risperidone are not mediated by blocking dopamine signaling through D1 or D2, but instead are similar to the effects induced by dopamine itself.

### Risperidone treatment did not appear to alter peripheral macrophages

**Figure 5:**
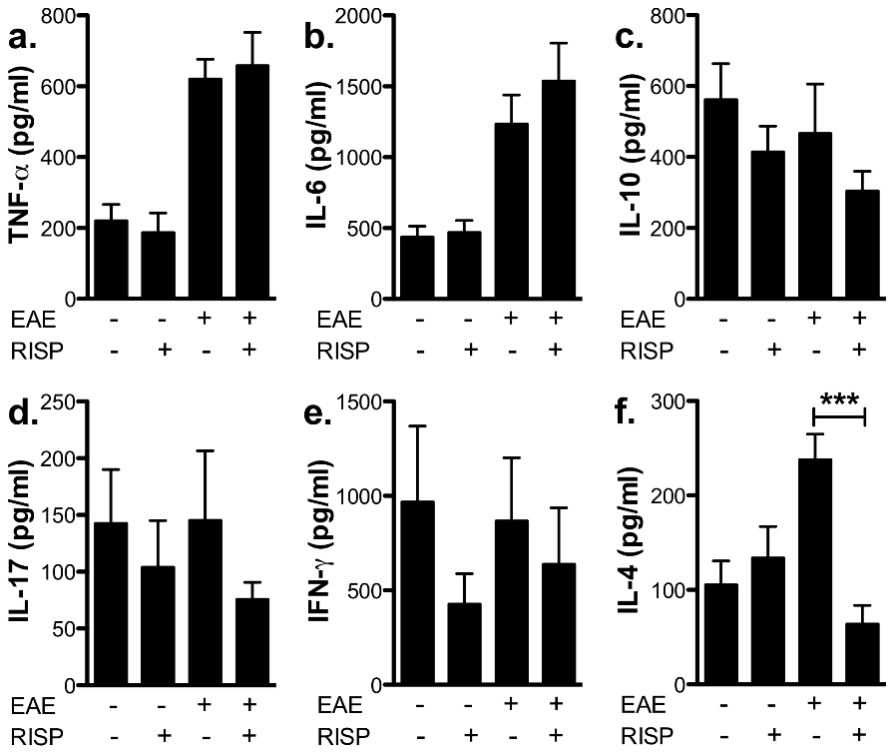
Treatment with risperidone *in vivo* does not alter LPS-stimulated splenic cytokine production. Splenocytes were isolated from risperidone- and vehicle-treated, unimmunized and immunized mice 15 days post-immunization and stimulated *in vitro* with LPS for 24 hours. TNF-α (**a**), IL-6 (**b**), IL-10 (**c**), IL-17a (**d**), IFN- γ (**e**), IL-4 (**f**) production from individual mice was assessed by CBA. Shown are the means and SEM of individual mice from three experiments (n = 10-15 per group). *** p<.05 by one-way ANOVA with Newman-Keul's multiple comparison test.

Because risperidone concentration-dependently reduced macrophage activation *in vitro*, we investigated whether any difference in macrophage activation by risperidone treatment could be detected *in vivo* during EAE. Since we believe that orally-administered risperidone should be acting in a systemic manner and because the spleen is a rich source of several macrophage populations, we investigated how risperidone treatment altered the response of splenocytes to the LPS and characterized their ex *vivo* phenotype. In contrast to the short term *in vitro* macrophage cultures, splenocytes isolated from immunized mice treated with risperidone *in vivo* for 15 days produced similar levels of pro-inflammatory cytokines as vehicle-treated, immunized mice in response to LPS (Figure 5). Similar results were found in unimmunized mice (Figure 5). While there was no effect on IL-10 production, it is interesting to note that IL-4 levels were elevated by immunization and this elevation was not found in splenocyte cultures from risperidone-treated, immunized mice (Figure 5). However, overall these results indicate that *in vivo*, chronic risperidone treatment does not significantly alter the ability of splenocytes to respond to an innate activator such as LPS.

**Figure 6:**
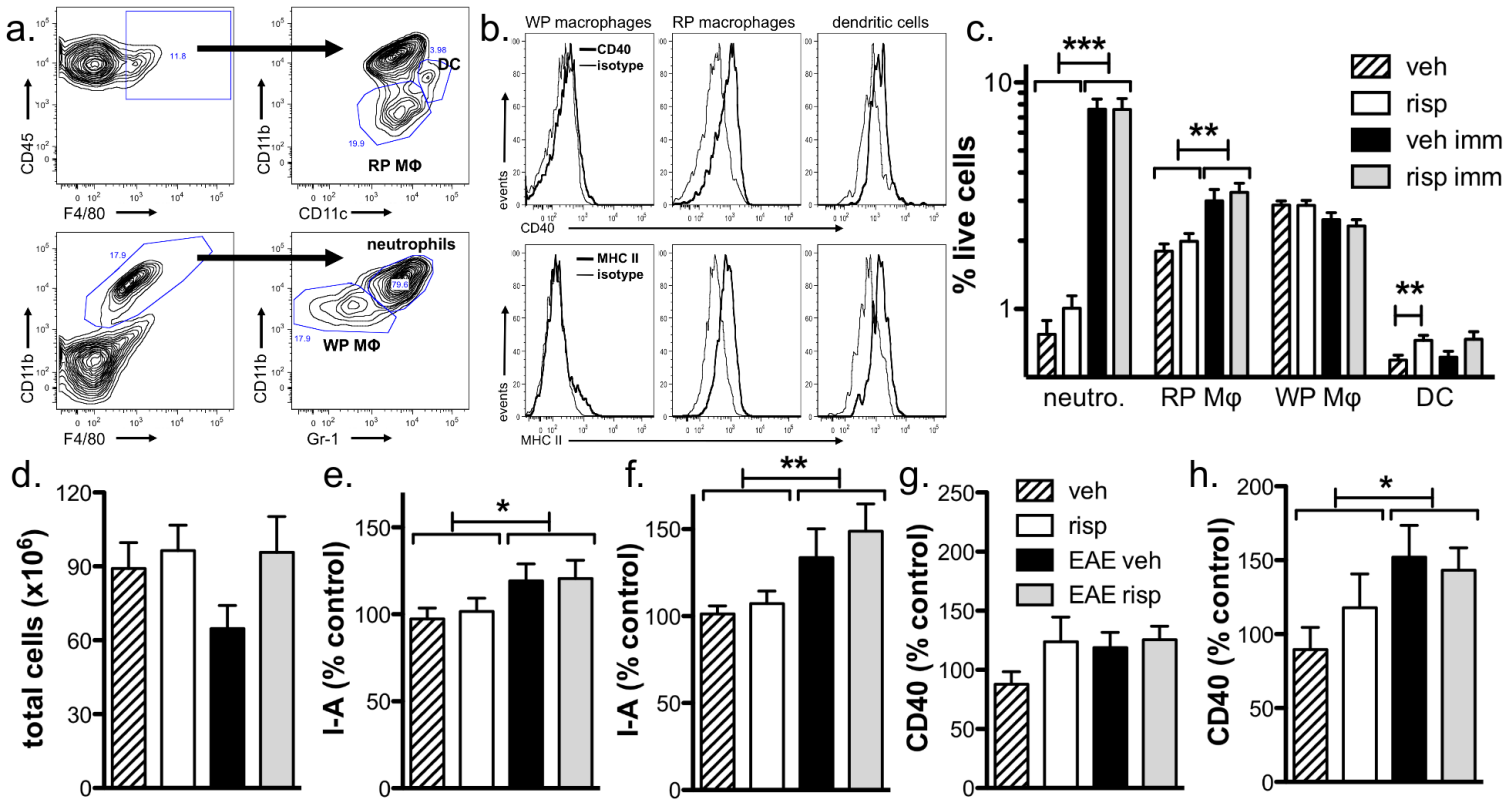
Risperidone treatment does not change the percentage or activation of splenic myeloid populations during EAE. **a.** Gating strategy for the major splenic myeloid populations. **b.** Red pulp macrophages (RP MΦ) and DC but not white pulp macrophages express MHC II and CD40. Shown are representative plots from a vehicle-treated, immunized mouse. **c.** Immunization induces an increase in neutrophils and red pulp macrophages and risperidone alone enhances splenic DC. Splenocytes were isolated from risperidone- and vehicle-treated, unimmunized and immunized mice 15 days post-immunization and gated as shown in **a**. Shown are the means and SEM of individual mice from three experiments (n = 10–15 per group). **p<0.01 and ***p<.001 by one-way ANOVA with Newman-Keul's multiple comparison test. **d.** No difference in the number of total splenocytes is observed. Splenocytes were isolated from risperidone- and vehicle-treated, unimmunized and immunized mice 15 days post-immunization and live cells counted by the trypan blue exclusion assay. Shown are the means and SEM of individual mice from three experiments (n = 10–15 per group). **e–f.** Immunization increases the expression of I-A on RP MΦ (**e**) and DC (**f**) and CD40 on DC (**h**) and risperidone does not alter these levels. Splenocytes were isolated from risperidone- and vehicle-treated, unimmunized and immunized mice 15 days post-immunization and gated as shown in **a**. The expression (ΔMFI compared to isotype controls) of I-A and CD40 are expressed as % of vehicle-treated, unimmunized group. Shown are the means and SEM of individual mice from three experiments (n = 10–15 per group). *p<0.05 and **p<0.01 by two-way ANOVA to distinguish overall drug and immunization effects.

When splenic myeloid populations were assessed by flow cytometric analysis (Figure 6a), we found that immunization led to an increase in the percentage of neutrophils and red pulp macrophages (Figure 6a and c) and risperidone treatment did not modify these populations (Figure 6c). White pulp macrophages were not affected by immunization or risperidone treatment but the percentage of DC was found to be enhanced by risperidone in unimmunized mice (Figure 6c). Furthermore, neither immunization nor risperidone treatment altered the total number of splenocytes (Figure 6d) nor the total number in each of the myeloid populations (data not shown).

To determine if drug treatment altered the activation of these myeloid populations, I-A (i.e. MHC class II) and CD40 expression were assessed, and these markers were found to be expressed primarily on DC and red pulp macrophages (Figure 6b). As expected, I-A was upregulated on DC and red pulp macrophages from immunized mice; however, risperidone-treatment did not alter I-A expression in these populations from unimmunized or immunized mice (Figure 6e and f). Additionally, while immunization upregulated CD40 on DC but not red pulp macrophages (Figure 6g and h), we did not observe any significant effect on CD40 expression by risperidone treatment (Figure 6g and h). Taken together, these findings suggest that risperidone treatment does not exert major immunomodulatory effects on peripheral myeloid cells in this system.

### Risperidone treatment significantly reduced the activation of microglia and macrophages in the CNS during EAE

**Figure 7:**
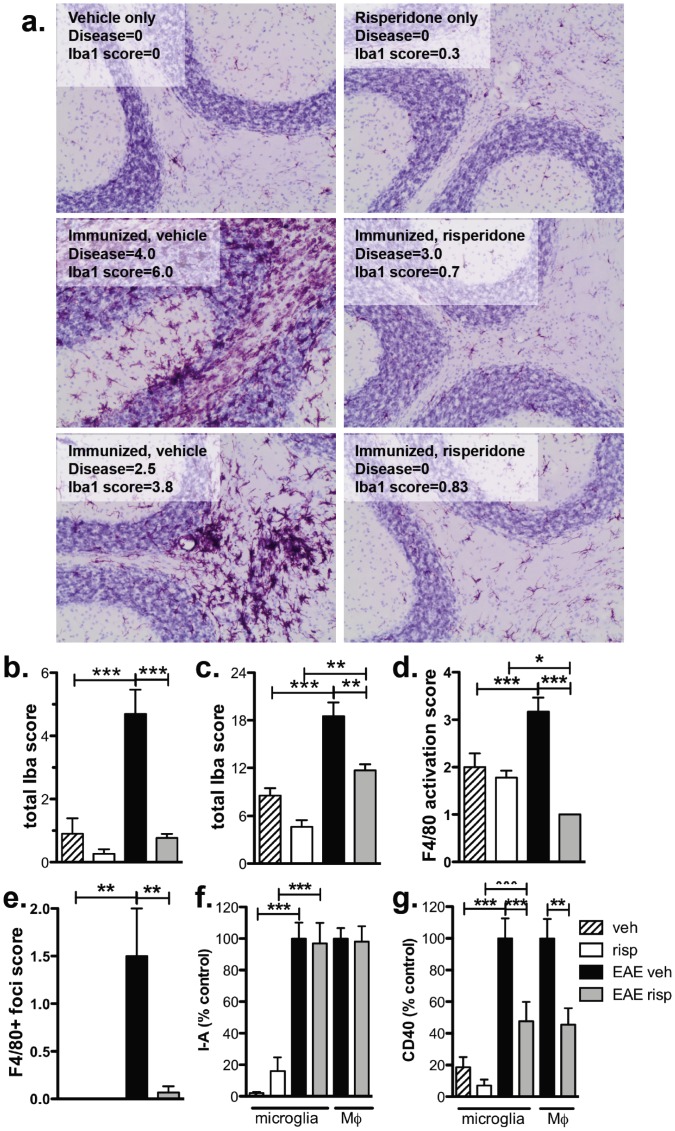
Risperidone treatment significantly reduces microglial activation in the CNS of immunized mice during EAE. **a.** Iba-1 expression (deep pink) in the cerebellum was assessed by immunohistochemistry and counterstained with hematoxylin (light purple). Shown are representative sections from unimmunized and immunized, vehicle and risperidone-treated mice as well as the “Iba-1 score” and “disease score” at time of euthanasia. **b & c.** Iba-1 expression in the cerebellum (**b**) and all brain and spinal cord regions (**c**) assessed (cerebellum, hippocampus, brain stem, olfactory bulb, and spinal cord). Shown are the means and SEM of individual mice from one of two experiments (n = 3 per unimmunized group; 4–5 per immunized group). **p<0.01 and ***p<0.001 by one-way ANOVA with Newman-Keul's multiple comparison test. **d & e.** Risperidone reduces the level of F4/80 (**d**) and the number of F4/80+ foci (**e**) in the cerebellum of immunized mice compared to vehicle treatment. F4/80 expression in the cerebellum was assessed by immunohistochemistry and shown are the means and SEM of individual mice (3 section per mouse) from 3 per unimmunized group and 4–5 per immunized group. * p<0.05, **p<0.01, and ***p<0.001 by one-way ANOVA with Newman-Keul's multiple comparison test. **f & g.** Risperidone reduces the expression of I-A (**f**) and CD40 (**g**) on microglia and macrophages in the CNS. CD45+ cells were isolated from the spinal cords of risperidone- and vehicle-treated, unimmunized and immunized mice 15 days post-immunization and the expression of I-A (**f**) and CD40 (**g**; ΔMFI compared to isotype controls) expressed as % of vehicle-treated, immunized group. Shown are the means and SEM of individual mice from three experiments (n = 10–15 per group). **p<0.01 and ***p<0.001 by one-way ANOVA with Newman-Keul's multiple comparison test (microglia) or unpaired Student's t test (macrophages) compared to vehicle-treated, immunized group.

Previous studies have shown that risperidone can reduce LPS-stimulated pro-inflammatory cytokine production by microglia *in vitro* [22]. To identify if risperidone treatment was able to alter microglial activation *in vivo*, the expression of Iba-1, a microglial and macrophage marker, was assessed in brain and spinal cord sections of unimmunized and immunized mice treated with risperidone or vehicle alone. Because T cell-rich inflammatory lesions occur in the spinal cords but not the upper CNS 15 days post immunization in C57BL/6 mice, the expression of Iba-1 in the upper CNS (e.g. cerebellum, hippocampus) represents the activation of the resident immune cells (i.e. microglia and perivascular macrophages) whereas the Iba-1 expression in the spinal cord will include both resident microglia and infiltrating macrophages at this time point. Figure 7a illustrates the pattern of Iba-1 expression found in the cerebellum of mice in each treatment group and clearly shows the presence of individual microglia with a “ramified” morphology in all groups as well as foci of Iba-1^+^ cells in the immunized, vehicle-treated mice. To take into account both the expression level as well as the expression pattern, an Iba-1 score was used that is the sum of the score for level of expression (0; normal - 3; intense) and the score for the number of Iba-1^+^ foci (0; none – 3; 7+ foci; See Figure S1a for scoring details and examples).

Using this scoring method, we found a significant increase in the expression of Iba-1 on microglia in the cerebellum and brain stem of immunized mice, and this expression was significantly reduced by risperidone treatment (Figure 7b and Figure S1c). In contrast to the cerebellum and brain stem, Iba-1 was not significantly reduced on microglia in the hippocampus, olfactory bulb, or spinal cord of risperidone-treated, immunized mice suggesting that risperidone treatment may be more effective at reducing microglial activation in specific brain regions (Figure S1c). Combining the scores in all 5 regions examined (i.e. cerebellum, hippocampus, brain stem, olfactory bulb, and spinal cord), a significant reduction in Iba-1 expression was found in risperidone-treated, immunized animals (Figure 7c). Finally, we assessed F4/80 expression of microglia and macrophages in the upper CNS regions (Figure S1b) and found a reduction in the level of expression and number of F4/80+ foci in risperidone-treated, immunized mice compared to vehicle-treated, immunized mice in the cerebellum (Figure 7d and e), brain stem and hippocampus (data not shown). As with Iba-1 expression, the greatest effects were observed in the cerebellum.

To determine whether risperidone treatment altered the activation of resident microglia and/or infiltrating macrophages, we isolated CD45+ cells from the spinal cords of vehicle- or risperidone-treated mice 15 days after immunization when significant inflammatory lesions occur and compared the expression of I-A and CD40 on microglia and macrophages (Figure S5 for gating strategy). While only low levels of I-A and CD40 were detected on microglia from unimmunized mice, these levels were enhanced by immunization and were similar to the levels expressed on macrophages in the CNS of immunized mice (Figure 7 f and g). The expression of these markers on macrophages in the spinal cords of unimmunized mice was not determined due to the low number of macrophages present in the absence of inflammation (Figure S5a). Similar to the effects on Iba-1 expression, risperidone treatment led to a significant reduction in the expression of CD40 on both microglia and macrophages from immunized mice although no change in I-A was detected (Figure 7f and g). In conclusion, these data indicate that risperidone treatment has direct immunomodulatory effects on both macrophages and microglia in the CNS but not on peripheral macrophage populations. Furthermore, taken together our results suggest that this myeloid-specific immunomodulation may be involved in the reduction of disease by the atypical antipsychotic agent, risperidone, through a unique mechanism independent of blocking dopamine signals through D1 or D2.

## Discussion

Because the atypical antipsychotic agent, risperidone, has been shown to modulate the immune system both peripherally and within the CNS, we investigated the ability of risperidone to modify disease in EAE, an animal model of MS. EAE immunized mice that were treated with risperidone had decreased disease severity and reduced cumulative disease. These mice also exhibited fewer spinal cord lesions and attenuated antigen-specific Th17 responses, during the peak but not chronic phase of disease. Additionally, the administration of another atypical antipsychotic agent, clozapine, had similar effects *in vitro* and *in vivo* suggesting that a common underlying mechanism of action may mediate these immunomodulatory effects. Moreover, the *in vitro* and *in vivo* effects of risperidone do not appear to be due to blocking of dopamine signaling through D1 or D2 receptors, and the *in vitro* effects of risperidone are similar to the effects of dopamine itself. Finally, we found that the most significant immunomodulatory effects occurred in the CNS and not the periphery as we originally expected, suggesting that the protective immunomodulatory effects of risperidone treatment may be preferentially targeted to the areas most relevant to MS.

While no studies have explored the use of the atypical antipsychotic drugs risperidone or clozapine in the context of autoimmunity, it has been demonstrated that atypical antipsychotic agents such as risperidone, clozapine and olanzapine have anti-inflammatory effects in induced inflammation models [7], [9], [22], [23] The mechanisms of action with which these drugs alter inflammatory responses are ambiguous; however, as many immune cells respond to neurotransmitter signalling, the antagonism of neurotransmitter receptors by antipsychotics is a likely cause of these effects. Leukocytes express receptors for many neurotransmitters including dopamine, serotonin, glutamate, acetylcholine and catecholamines [40]. Additionally, it has been demonstrated that neurotransmitters including dopamine and serotonin can be synthesized and/or released by dendritic cells, macrophages and T cells [39], [41], [42], [43], [44], [45].

Previous work by Nakano *et al*. demonstrated that blockade of dopamine D1 type receptors using the antagonist SCH23390 resulted in the inhibition of EAE while in contrast, the D2 receptor antagonists L750667 and sulpiride increased EAE severity [46]. Likewise administration of the dopamine D2 agonist bromocriptine following EAE onset reduced severity and duration of disease symptoms [47]. From these studies, we would expect that risperidone, a potent antagonist of dopamine D_2_ receptors, would enhance not reduce EAE disease expression and thus the observed protection seen in the current study may appear contradictory to these previous findings. However, the difference may be due to the range of different receptors that are antagonized by atypical antipsychotics as opposed to the more selective antagonists used by Nakano *et al*. Additionally, as risperidone can also antagonize D1, albeit not to the same affinity as D2, it is possible that the protective effect through D1 is more potent than the detrimental effects mediated by D2 antagonism. However, our *in vitro* studies assessing the effect of D1 and D2 antagonism and dopamine on IL-12 production by macrophages suggest that the anti-inflammatory activity of risperidone is not mediated by blockade of dopamine signaling at D1 or D2.

While risperidone has high affinity for D2 and to a lesser extent D1, it also potently antagonizes serotonin 5HT_2_ receptors, other dopamine and serotonin receptors, histamine H_1_ and adrenergic α_1_ receptors at low nanomolar concentrations [14], [15], [20]. Previous research has shown that the administration 5-HT_1A_/D_4_ antagonist WAY100635 was found to suppress disease expression, while the absence of 5-HT transporters resulted in decreased CNS inflammatory infiltrates [13], [48], [49]. Additionally, administration of prazosin, an α1-adrenergic antagonist, or hydroxyzine, an H_1_ histamine receptor antagonist, reduces EAE disease severity and progression. It is therefore conceivable that the disease modifying effects of risperidone, observed in the current study, could be mediated through antagonism of a number of different neurotransmitter receptors. Furthermore, the immunoregulatory properties of dopamine alone suggest that signaling by histamine, serotonin, and adrenaline may not be important immunomodulatory signals in this system. Instead, when taken together these studies suggest that by inhibiting the binding of dopamine to its receptors, dopamine may be free to bind receptors of much lower affinity such as the β2-adrenergic receptor. Previous work by Hasko *et al* showed that the effects of dopamine could be suppressed by a β2-adrenergic receptor antagonist suggesting that dopamine can also act through this receptor [50]. Whether the protective effects of risperidone during EAE are mediated by dopamine signaling though β2-adrenergic receptors remain to be determined.

Several studies have reported immunomodulatory effects by antipsychotic agents on microglia activation and cytokine expression; for example, Kato *et al*. showed that IFN-γ-stimulated 6-3 microglial cells treated with risperidone had reduced NO, IL-1β, IL-6 and TNF-α cytokine expression [8], [35], [36]. We have demonstrated that risperidone and clozapine can also cause reductions in NO and IL-12p40 production by LPS-stimulated macrophages, further supporting the hypothesis that these atypical antipsychotic agents can have a direct effect on cells of a myeloid linage. In addition, we found a concurrent increase in IL-10 production by risperidone-treated macrophages, which indicates that this drug causes immunomodulation not simply immunosuppression. This finding is of particular relevance as while MS and EAE are considered primarily T cell-mediated diseases, macrophages play a crucial role in the disease induction and progression [51], [52]. Indeed, it has been shown that altering macrophage/monocyte activation within an inflammatory environment towards an anti-inflammatory phenotype can inhibit EAE disease induction or reduce disease severity [25], [53], [54]. However, while we found that risperidone treatment reduced the activation of microglia and macrophages in the CNS *in vivo*, we did not find any significant effects on the splenic myeloid populations or responses. Thus, our findings suggest that the atypical antipsychotic agent risperidone is more potent at inducing an anti-inflammatory phenotype in the CNS than in the periphery, and this site-specific action may contribute to its ability to reduce EAE severity. Given that we found the greatest effects in the cerebellum and not the spinal cord, which is the predominant lesion site in the C57BL/6 EAE model [34], it is very possible that risperidone would have much greater protective effects in MS patients who have significant cerebellar involvement [55]. Finally, our observation that microglial activation in the upper CNS and in the cerebellum, in particular, is dramatically reduced in immunized animals treated with risperidone further supports our theory that modification of myeloid responses, directly or indirectly, is the main mechanism of action of these agents.

While modifying myeloid responses may be the main mechanism of action, it is also possible that alterations in peripheral T cell responses are also a contributing factor. The modest shift in the balance of antigen specific responses to favor Th1 over Th17 or Th2 provides evidence of this possibility; however, from our *in vitro* model of macrophage-mediated Th1 cell activation, it is clear that risperidone can also impair Th1 biasing and this impairment is due to the direct effect of risperidone on the macrophage. While this *in vitro* model provides evidence that Th1 as well as Th17 and Th2 biasing can be affected by risperidone, it is important to consider that in the more Th17-driven EAE model, IFN-γ can play a protective role by limiting disease severity [56], [57], and Th1 cells have been shown to be less encephalitogenic than Th17 cells [58] [59]. Thus, an increased ratio of IFN-γ to IL-17a-MOG_35-55_ reactive T cells in risperidone-treated mice may be beneficial in limiting the disease severity.

Collectively, our studies indicate that the atypical antipsychotic agent, risperidone, can alter EAE disease severity when administered at concentrations physiologically comparable to doses used clinically to treat schizophrenia [31]. While antigen-specific responses are shifted and risperidone can directly impair Th biasing, we believe that the primary effect on disease modification is due to risperidone's ability to cause a reduction in macrophage and microglial activation in the CNS. This is the first study to explore the effects of risperidone and another atypical antipsychotic agent (i.e. clozapine) in EAE, and to show that the anti-inflammatory effect is not mediated by blockade of dopamine signaling through D1 and D2. Our findings raise the possibility that these agents could provide therapeutic benefit in inflammatory diseases such as MS, and as risperidone and clozapine have been clinically used for the treatment of schizophrenia for a number of years, dosing, safety and side effect profiles are well understood. All these factors suggest further investigation into the therapeutic potential for the treatment of MS is warranted.

## Supporting Information

Figure S1 **Scoring method for Iba-1 (a) and F4/80 (b) in the cerebellum and total Iba-1 scores in the hippocampus, brain stem, olfactory bulb, and spinals cords of vehicle- or risperidone-treated, immunized or unimmunized mice.**
(DOCX)

Figure S2 **During the chronic phase of EAE, there is an expansion in splenic Treg numbers and increase in MOG-specific IFN-γ production by splenocytes.**
(DOCX)

Figure S3 **Spinal cord lesions are reduced by risperidone treatment.**
(DOCX)

Figure S4 **Bone marrow-derived macrophages (BMMΦ) express dopamine receptors D1 and D2 and exposure to risperidone alters the ability of BMMΦ to bias CD4 T cells.**
(DOCX)

Figure S5 **Identification (a) and assessment (b) of activation of microglia and macrophages in the spinal cord during EAE.**
(DOCX)
