# Supplementary material for: Treatment with the Antipsychotic Agent, Risperidone, Reduces Disease Severity in Experimental Autoimmune Encephalomyelitis
Source: PLoS One. 2014 Aug 12;9(8):e104430. doi: 10.1371/journal.pone.0104430 (PMC4130540; doi:10.1371/journal.pone.0104430)
Supplement: Figure S1 — Scoring method for Iba-1 (a) and F4/80 (b) in the cerebellum and total Iba-1 scores in the hippocampus, brain stem, olfactory bulb, and spinals cords of vehicle- or risperidone-treated, immunized or unimmunized mice. (DOCX) [file pone.0104430.s001.docx]

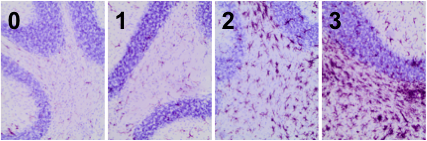


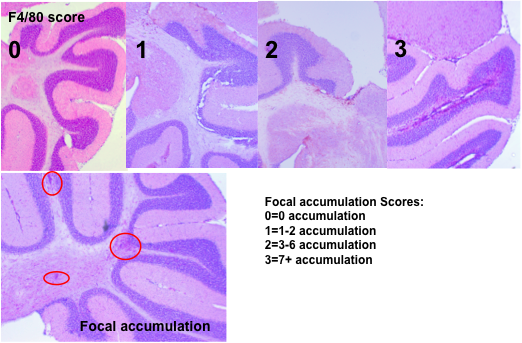


**b.**

**a.**


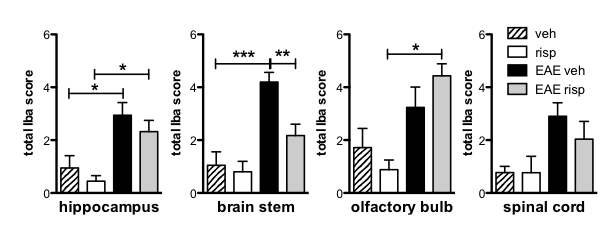
Figure S1: Scoring method for Iba-1 **(a)** and F4/80 **(b)** in the cerebellum and total Iba-1 scores in the hippocampus, brain stem, olfactory bulb, and spinals cords of vehicle- or risperidone-treated, immunized or unimmunized mice. **a.** Iba-1 expression (deep pink) in the cerebellum was assessed by immunohistochemistry and counterstained with hematoxylin (light purple). Shown are representative tissue sections from the cerebellum indicative of the 0-3 score for Iba-1 intensity. **b.** F4/80 expression (deep pink) in the cerebellum was assessed by immunohistochemistry and counterstained with hematoxylin (light purple). Shown are representative tissue sections from the cerebellum indicative of the 0-3 score for F4/80 intensity (top). The focal accumulation scoring system (bottom) shows foci (red circles) and # foci for each score. **c.** Total Iba-1 (0-6) in the hippocampus, brain stem, olfactory bulb, and spinal cord regions was assessed by immunohistochemistry. Shown are the means and SEM of individual mice from one (spinal cord) or two (all other) experiments (n= 6 per unimmunized group; 9-10 per immunized group). *p < 0.05, **p < 0.01, and ***P < 0.001 by one-way ANOVA with Neuman-Keuls Multiple Comparison Test.

**c.**
