# Supplementary material for: Treatment with the Antipsychotic Agent, Risperidone, Reduces Disease Severity in Experimental Autoimmune Encephalomyelitis
Source: PLoS One. 2014 Aug 12;9(8):e104430. doi: 10.1371/journal.pone.0104430 (PMC4130540; doi:10.1371/journal.pone.0104430)
Supplement: Figure S2 — During the chronic phase of EAE, there is an expansion in splenic Treg numbers and increase in MOG-specific IFN-γ production by splenocytes. (DOCX) [file pone.0104430.s002.docx]

**
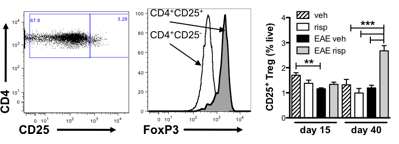
**
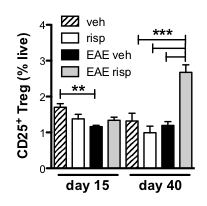


**a.**


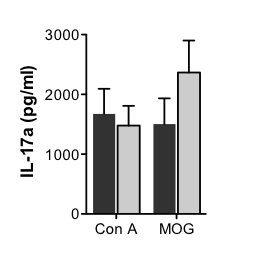

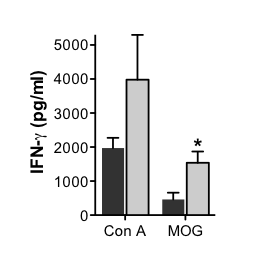

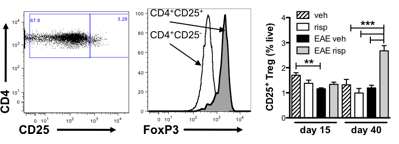


**b.**

**c.**

**d.**

Figure S2: During the chronic phase of EAE, there is an expansion in splenic Treg numbers and increase in MOG-specific IFN-γ production by splenocytes. Mice were treated with risperidone (3 mg/kg/day) or vehicle in their drinking water from the time of immunization. (a) Splenic Tregs (CD25^+^CD4^+^) were assessed by flow cytometry 15 and 40 days post immunization and were confirmed to express FoxP3. Shown are representative plots from a vehicle-treated, unimmunized mouse and the means and SEM of individual mice (n = 10-15 per group). *p < 0.05, **p < 0.01, and ***p < 0.001 by one-way ANOVA with Bonferroni’s multiple comparison test. (b-d) Splenocytes were isolated 40 days post immunization and stimulated *in vitro* with MOG peptide for 72 hours. IL-17a (b), IFN-γ (c), and IL-10 (d) were measured in culture supernatants by ELISA. Shown are the means and SEM of individual mice. *p < 0.05 by unpaired Student’s t test.
