# Supplementary material for: Treatment with the Antipsychotic Agent, Risperidone, Reduces Disease Severity in Experimental Autoimmune Encephalomyelitis
Source: PLoS One. 2014 Aug 12;9(8):e104430. doi: 10.1371/journal.pone.0104430 (PMC4130540; doi:10.1371/journal.pone.0104430)
Supplement: Figure S3 — Spinal cord lesions are reduced by risperidone treatment. (DOCX) [file pone.0104430.s003.docx]

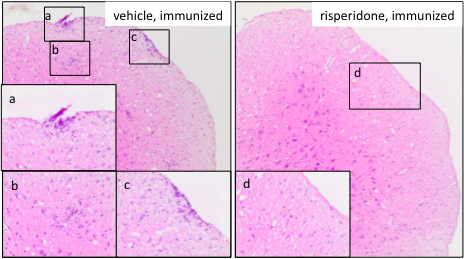


Figure S3: Spinal cord lesions are reduced by risperidone treatment. Mice were treated with risperidone (3 mg/kg/day) or vehicle in their drinking water from the time of immunization, and spinal cords were collected for analysis 15 days post immunization. Shown are representative H & E stained tissue from vehicle-treated (left) and risperidone-treated (right) mice. Boxes a-c highlight inflammatory infiltrates while Box d shows the absence of these infiltrates with risperidone treatment.
