# Supplementary material for: Treatment with the Antipsychotic Agent, Risperidone, Reduces Disease Severity in Experimental Autoimmune Encephalomyelitis
Source: PLoS One. 2014 Aug 12;9(8):e104430. doi: 10.1371/journal.pone.0104430 (PMC4130540; doi:10.1371/journal.pone.0104430)
Supplement: Figure S4 — Bone marrow-derived macrophages (BMMΦ) express dopamine receptors D1 and D2 and exposure to risperidone alters the ability of BMMΦ to bias CD4 T cells. (DOCX) [file pone.0104430.s004.docx]

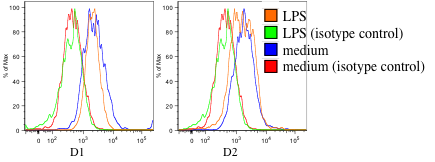


**a.**


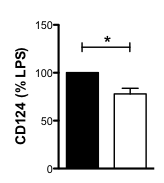

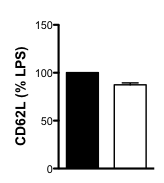

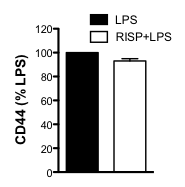

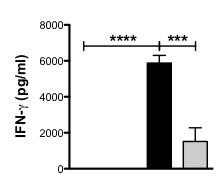

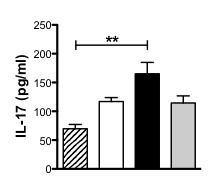

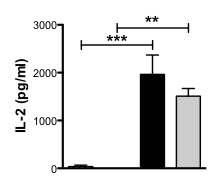

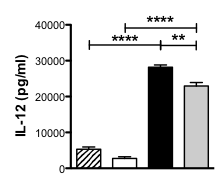

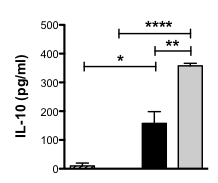

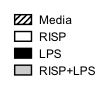


**b.**

**c.**

Figure S4: Bone marrow-derived macrophages (BMMΦ) express dopamine receptors D1 and D2 and exposure to risperidone alters the ability of BMMΦ to bias CD4 T cells. **a.** After 24-hour culture in the presence or absence of LPS (200 ng/ml), BMMΦ had detectable levels of D1 and D2 as assessed by flow cytometry. Shown are representative plots of all live cells comparing anti-D1 and anti-D2 antibodies (Calbiochem; rabbit anti-mouse D1 or D2 antibodies) to isotype control antibodies. **b.** BMMΦ were primed overnight with IFN-γ and stimulated with LPS (200 ng/ml) in the presence or absence of risperidone (50 μM) for 4 hours prior to the addition of purified 2D2 CD4 T cells and MOG peptide (50 μg/ml). After 72 hours, supernatants were isolated, and cytokines assessed by ELISA. Shown are the means and SEM of triplicate wells from one representative experiment of four experiments total. *p < 0.05, **p < 0.01, ***p < 0.001, and ****p<0.0001 by one way ANOVA with Newman-Keul’s multiple comparison post test. **c.** BMMΦ and T cells were cultured as in Suppl. Fig. 2b, and CD124, CD62L, and CD44 expression was assessed on the T cells after 72 hours by flow cytometry. Shown are the means and SEM from three experiments. *p < 0.05 by one way ANOVA with Newman-Keul’s multiple comparison post test.
