# Supplementary material for: Treatment with the Antipsychotic Agent, Risperidone, Reduces Disease Severity in Experimental Autoimmune Encephalomyelitis
Source: PLoS One. 2014 Aug 12;9(8):e104430. doi: 10.1371/journal.pone.0104430 (PMC4130540; doi:10.1371/journal.pone.0104430)
Supplement: Figure S5 — Identification (a) and assessment (b) of activation of microglia and macrophages in the spinal cord during EAE. (DOCX) [file pone.0104430.s005.docx]

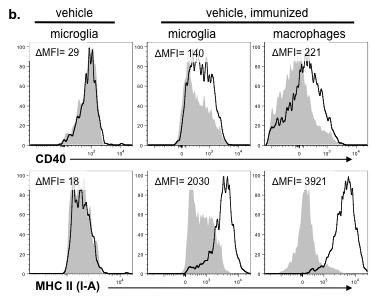

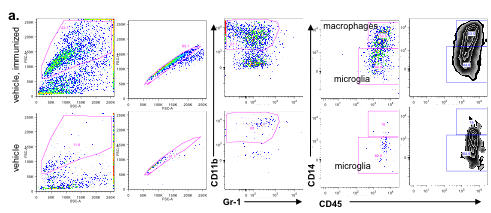


Figure S5: Identification (a) and assessment (b) of activation of microglia and macrophages in the spinal cord during EAE. **a.** Gating strategy showing gating for live, single cells using FSC and SSC followed by CD11b^+^ but Gr-1^-^ gating for macrophages and microglia. CD14^+^CD45^high^ were considered macrophages and CD14^-^CD45^int^ were considered microglia. Shown are representative plots from vehicle-treated, immunized (top) and unimmunized (bottom) mice. **b.** Representative plots indicate that microglia and macrophages express CD40 (top) and I-A (bottom) after but not before immunization. Dark lines indicate specific staining while the shaded histograms indicate the isotype control for this population. The change in MFI is shown for each plot (MFI of specific antibody – MFI of isotype).
